# E3 ubiquitin ligases and deubiquitinases in bladder cancer tumorigenesis and implications for immunotherapies

**DOI:** 10.3389/fimmu.2023.1226057

**Published:** 2023-07-11

**Authors:** Maoyu Wang, Zhensheng Zhang, Zhizhou Li, Yasheng Zhu, Chuanliang Xu

**Affiliations:** ^1^ Department of Urology, Shanghai Changhai Hospital, Naval Medical University, Shanghai, China; ^2^ Department of Urology, Shanghai Changzheng Hospital, Naval Medical University, Shanghai, China

**Keywords:** bladder cancer, E3 ubiquitin ligase, deubiquitinases, immunotherapy, tumorigenesis

## Abstract

With the rapidly increasing incidence of bladder cancer in China and worldwide, great efforts have been made to understand the detailed mechanism of bladder cancer tumorigenesis. Recently, the introduction of immune checkpoint inhibitor-based immunotherapy has changed the treatment strategy for bladder cancer, especially for advanced bladder cancer, and has improved the survival of patients. The ubiquitin–proteasome system, which affects many biological processes, plays an important role in bladder cancer. Several E3 ubiquitin ligases and deubiquitinases target immune checkpoints, either directly or indirectly. In this review, we summarize the recent progress in E3 ubiquitin ligases and deubiquitinases in bladder cancer tumorigenesis and further highlight the implications for bladder cancer immunotherapies.

## 1 Introduction

Bladder cancer (BCa) is one of the most common types of cancer, with 550,000 new cases and 200,000 deaths annually (1). While the 5-year survival rate of all bladder cancer patients is 77.1%, the rate drops dramatically to 36.3% for regional disease and 4.6% for metastatic disease (2). Therefore, adjunctive therapy is needed to improve the prognosis of invasive and metastatic diseases. Cisplatin and gemcitabine combination chemotherapy has been applied for advanced bladder cancer (3); however, no major improvements in survival rate have been achieved until recently. The 5-year survival rate for patients with metastasis is 15% (3).

Immunotherapy, especially immune checkpoint inhibitors, is widely used for the treatment of different cancers (4, 5). BCa has been reported to be relatively sensitive to immunotherapy (6, 7). In May 2016, atezolizumab was the first PD-L1 inhibitor approved by the Food and Drug Administration (FDA) for bladder cancer (8). Since then, another four immune checkpoint inhibitors targeting PD-1 or PD-L1 for locally advanced and metastatic bladder cancer, including Nivolumab, Pembrolizumab, Avelumab, and Durvalumab have been approved by FDA for bladder cancer (8–10). However, owing to a lack of response, only a small group of patients with BCa can benefit from these agents (11). Taking PD-L1 as example, many studies have verified that PD-L1 expression is correlated with anti-PD-1/PD-L1 treatment, where high PD-L1 expression is equal to a good response to anti-PD-1/PD-L1 treatment (12). Thus, exploring the mechanism and identifying other reagents that can improve the efficacy of immune checkpoint blockade (ICB) is urgently needed (13). A series of mechanisms of PD-L1 regulation by post-translational modifications have been revealed in different cancers among recent research, including bladder cancer (14–16).

Ubiquitination and deubiquitinating modifications are highly conserved posttranslational modifications (PTMs) in mammals that play important roles in many biological processes and diseases, including cancers. The ubiquitin-activating enzyme E1, ubiquitin-conjugating enzyme E2, and ubiquitin ligase E3 contribute to the step-by-step process of ubiquitination. Ubiquitination involves the transfer of the C-terminal glycine of ubiquitin to the -NH2 group of the substrate lysine residue. Monoubiquitination, multiubiquitination, and polyubiquitination, which lead to proteolysis and signal transduction, are the three main types of ubiquitination (17). On the other hand, deubiquitinases (DUBs) can reverse ubiquitination by removing ubiquitin chains, thereby preserving the expression of the substrate protein while preventing ubiquitination. Most elements of biological activity depend on the interplay between ubiquitination and deubiquitination (13).

Numerous studies have demonstrated that the ubiquitin proteasome system (UPS) is related to the occurrence and progression of bladder cancer and that E3 ubiquitin ligases may be promising therapeutic targets (18–21). Meanwhile, the interaction between ubiquitination modification and immune-related molecules is emerging as a crucial regulatory mechanism and has recently draws great research interest (16, 22–25).

In this review, we summarize recent findings on protein ubiquitination and deubiquitinating enzymes in bladder cancer tumorigenesis and progression, as well as recent advances in the regulation of cancer immunotherapy effects.

## 2 Roles and mechanisms of E3 ubiquitin ligases in bladder cancer

### 2.1 The category of E3 ubiquitin ligases

Over 600 types of E3 ubiquitin ligases involved in the degradation of proteins have been discovered in humans (26). E3 ligases are classified into three subtypes: the interesting new gene (RING)-type, the homologous to E6AP carboxyl terminus (HECT)-type, and the RING-between-RING (RBR)-type (27). RING E3 ligases contain multiple subtypes, including monomers (c-CBL,E4B), homodimers (cIAP, CHIP), heterodimers (MDM2-MDMX), cullin-RING ligases (CRLs), and other RING E3s (28). CRLs are comprised of multiple subunits, which consist of four components: a cullin (CUL1,2,3,4A,4B,5,7,9), an adaptor protein, a substrate-recognizing receptor, and one RING protein (29–32). Moreover, SCF is the largest complex, consisting of SKP1, Cullin1, RBX1, and F-box proteins (29, 33). HECT structures are divided into three subfamilies: NEDD4 subfamily, HERC subfamily, and other HECT E3 ligases (34). RBRs are grouped into the Ariadne family and other RBRs (35). In particular, E3 ubiquitin ligases determine substrate specificity in the ubiquitination process.

### 2.2 Roles of E3 ubiquitin ligases in bladder cancer

In addition to maintaining the balance of intracellular proteins, E3 ligases are involved in multiple non-degradable functions including intracellular transport, autophagy, DNA damage repair, and metabolism (36). Thus, E3 ubiquitin ligases are critical for cellular processes. Therefore, their dysregulation may have a potential effect on the pathogenesis of cancer. Disorders of E3 ligases result in aberrant activation or inactivation of signaling pathways and the accumulation of misfolded or dysfunctional proteins (37), which promotes the occurrence and progression of cancer.

Numerous E3 ligases have been reported to be involved in bladder cancer tumorigenesis. They are involved in the regulation of key molecules including PD-L1, PTEN, and p53 (**Table 1**). In this section, we provide a detailed description of each E3 ligase in bladder cancer.

**Table 1:** E3 ligases in bladder cancer tumorigenesis.

E3	Function	Substrate	Pathway	Reference
RNF126	Promoting/oncogene	PTEN	PI3K/AKT	(38, 39)
RNF144A	Promoting/oncogene	PD-L1		(40, 41)
NEDD4	Promoting/oncogene	PD-L1		(16, 42)
		KLF8	microRNA-132/NRF2	(43, 44)
		PTEN		(42)
RBX1	Promoting/oncogene	p-IκBα	NF-κB	(45)
		DEPTOR	mTOR	(46)
		SUFU	RBX1-SUFU-GLI2	(47)
cIAP2	DNA damage response	MRE11		(48, 49)
FBW7	Tumor suppressor	ZMYND8		(50)
		RhoGDIα	p65/PTEN/FBW7/RhoGDIα	(51)
TRAF4	Promoting/oncogene		BMP/SMAD	(21)
TRIM21	Promoting/oncogene	ZHX3		(52)
TRIM65	Promoting/oncogene	ANXA2		(53)
TRIM25	Promoting/oncogene	RBPJ	Notch1	(54)
TRIM26	Promoting/oncogene		AKT/GSK3β/β-catenin	(55)
CUL4B	Promoting/oncogene	H2AK119	CUL4B/miR-372/373/PIK3CA/AKT	(56)
TRIM38	Promoting/oncogene	GLUT1		(57)
RFWD3,HUWE1 MDM2,DTL	Promoting/oncogene			(58–61)

#### 2.2.1 RNF126

RNF126 is a RING domain E3 ligase. A group of RNF126 substrates has been identified, including frataxin (62–64), epidermal growth factor receptor (64), pyruvate dehydrogenase kinases (65) and insulin-like growth factor II receptor (66). RNF126 is highly expressed in various cancers and strongly associated with tumorigenesis, including bladder cancer (38, 67–69). In BCa, RNF126 directly binds to PTEN via its C-terminal containing the RING domain and promotes the polyubiquitination and degradation of PTEN through the proteasome pathway (38). *In vivo* and *in vitro* studies have demonstrated that PTEN acts as an anti-oncogene, and PTEN silencing is closely related to the poor prognosis of patients with BCa (70). RNF126 silencing stabilizes PTEN, which antagonizes PI3K/AKT signaling pathway (38, 39), and promotes cell proliferation and metastasis when activated.

Moreover, previous studies revealed that RNF126 promotes the repair of DNA double-strand breaks via NHEJ and HR through different mechanisms (71, 72). The Ku70-Ku80 heterodimer recognizes DNA double-strand breaks (DSBs) and recruits proteins responsible for DNA repair by non-homologous end joining (NHEJ). While prolonged retention of Ku70/80 at DSBs prevents the completion of DNA repair, RNF126 ubiquitylates Ku80 at DSBs and promotes Ku70/80 dissociation from DSBs. In contrast, RNF126 can ubiquitinate and quench RNF168 function in the DNA damage response (71). Cisplatin has been widely used as first-line treatment for patients with advanced BCa (73). Furthermore, cisplatin induces cell apoptosis by accumulating DNA double-strand breaks. RNF126 depletion markedly increases the effect of cisplatin in inducing apoptosis in BCa cells (38). It has also been reported that RNF126 can directly bind and regulate PTEN stability through polyubiquitination, making RNF126 an attractive target for augmenting cisplatin-based chemotherapy and regulating bladder cancer tumorigenesis.

#### 2.2.2 RNF144A

RNF144A belongs to the RBR E3 ubiquitin ligase family. Epigenetic depletion of RNF144A has been detected in numerous human cancers, including glioblastoma (74), breast cancer (75), and bladder cancer (40), indicating that RNF144A may act as a tumor suppressor. Previous studies have found that RNF144A is upregulated by various DNA-damaging agents (76) and further promotes cancer cell apoptosis of cancer cells by ubiquitinating and degrading DNA-PKcs and BMI1 (74, 77).

In a recent study, the basal-squamous subtype of bladder cancer has been found to express relatively low levels of RNF144A and high levels of immune checkpoint protein programmed cell death ligand-1(PD-L1) (41). The carboxyl-terminal region (aa 250–292) of RNF144A is responsible for its interaction with PD-L1, and RNF144A mainly targets glycosylated PD-L1 for degradation (40), further indicating a complex mechanism between protein ubiquitination and glycosylation.

#### 2.2.3 NEDD4

NEDD4 is a HECT family E3 ubiquitin ligase (78). Mounting evidence has demonstrated that NEDD4 participates in the tumorigenesis of human cancers, such as cervical cancer (79), hepatocellular carcinoma (80), and breast cancer (81). NEDD4 is highly expressed in bladder cancer and promotes tumor cell migration and invasion (42, 43). KLF8 acts as a transcription factor in the Sp/KLF family and stimulates and promotes migration of bladder cancer cells. Moreover, miR-132 is downregulated by KLF8, which is overexpressed in bladder cancer. NEDD4 is conformed to interact with KLF8 (44). In bladder cancer, NEDD4 depletion significantly downregulated endogenous KLF8 ubiquitination, which affected the K63-linked polyubiquitination of KLF8, while K48-linked polyubiquitination remained unchanged. NEDD4 intensifies the stability and transcriptional activity of KLF8 through ubiquitination and affects the miR-132/NRF2 axis, thereby promoting tumor progression (44).

The ubiquitin ligase activity of NEDD4 can be promoted by FGFR1 and EGFR activation via tyrosine phosphorylation of NEDD4 (82). Previous studies have demonstrated that there is relatively decreased expression of PD-L1 in bladder cancer with FGFR3 mutations or high expression (41, 83, 84). Jing et al. (16)have indicated that the activation of FGFR3 promoted NEDD4 binding and phosphorylation and it had been reported that NEDD4 can be phosphorylated to greatly improve its ubiquitination capacity. NEDD4 depletion using CRISPR/Cas9-sgRNA remarkably upregulated PD-L1 expression in bladder cancer cells. NEDD4 targets and catalyzes the K48-linked polyubiquitination of PD-L1. These results reveal that NEDD4 is a critical regulator of PD-L1 expression in bladder cancer upon FGFR3 activation. This study provides powerful evidence for the combination of anti-PD-1 antibody therapy and erdafitinib, a tyrosine kinase inhibitor of FGFR1–4 (16).

As mentioned earlier, PTEN acts as an oncogene in bladder cancer. NEDD4 regulates PTEN levels in several types of human cancers (85). In bladder cancer, PTEN levels were increased by NEDD4 silencing (42). NEDD4 downregulation inhibits cell proliferation and apoptosis. However, the precise mechanism by which NEDD4 regulates PTEN expression has not been fully elucidated.

#### 2.2.4 RBX1

The cullin/RING ubiquitin ligase (CRL)family is the largest UPS E3 family (86). RBX1 forms the catalytic core of CRL complexes with different Cullin subunits (87). RBX1 is widely reported to be associated with poor clinical prognosis and is highly expressed in many cancers, including bladder cancer. In particular, RBX1 expression is significantly higher in muscle-invasive BCa and positively correlated with epithelial–mesenchymal transition (EMT) via inhibition of mTOR kinase activity by accumulation of the cullin-RING ligase (CRL) substrate mTOR-inhibitory protein DEPTOR (46).

Moreover, RBX1 has been confirmed to be positively correlated with activation of the NF-κB signaling pathway and nuclear p65 expression (45). p65 plays a key role in the canonical NF-κB pathway and is inactive in the cytoplasm upon binding to IκBα. Upon receiving the relevant signals, IκBα is phosphorylated, which is then ubiquitinated and degraded. Finally, p65 enters the nucleus and activates gene transcription (88). Therefore, IκBα-p65 is a key regulatory factor in the NF-κB signaling pathway. Activation of the NF-κB signaling pathway promotes tumor progression (89). By enhancing p-IκBα ubiquitination and degradation, RBX1 activates NF-κB signaling, which promotes p65 nuclear translocation and causes the transcription of several metastasis-related target genes including matrix metalloproteinase 9 (MMP9), vascular cell adhesion molecule 1 (VCAM1), and urokinase-type plasminogen activator receptor (uPAR) (45). Recently, Wang et al. demonstrated that RBX1 can activate the hedgehog pathway through the ubiquitinate suppressor of fused homolog (SUFU) for degradation, and dysregulation of the RBX1–SUFU–GLI2 axis play a pivotal role in bladder cancer progression (47).

#### 2.2.5 cIAP2

IAP family members have been indicated to act as a key role in the regulation of NF-κB signaling and participate in intrinsic and extrinsic cell death pathways (90). cIAP2 is a RING-type E3 ligase in the IAP family and has been demonstrated to play a pivotal role in DNA repair (91, 92). Although the expression of cIAP1 examined by immunohistochemical testing is highly correlated to bladder cancer TNM stage, tumor grade, disease recurrence, and tumor-related death (93) and cIAP2 precise function and substrate specificity is unclear, previous studies have a common sense that there is redundancy between cIAP1 and cIAP2 in the regulation of cell death (94, 95). Recently, cIAP2 was reported to be involved in regulating radiosensitization in bladder cancer (48).

Histone deacetylase (HDAC) inhibitors exhibit low toxicity in normal cells, and panobinostat, an HDAC inhibitor, is a promising radiosensitizer (96). Panobinostat downregulates MRE11 (49), which is a key player in DNA repair, leading to a decreased ability to repair DNA, thereby enhancing radio sensitization. In T24 cells, transfecting cIAP2 into cells in increasing quantities, a growing decrease in MRE11 levels was observed. cIAP2 downregulates MRE11 via proteasomal pathways and increases the ubiquitination of MRE11. Furthermore, T24 cells became more radiosensitive after panobinostat treatment when cIAP2 was silenced.

#### 2.2.6 FBW7

F-box and WD repeat domain-containing 7(FBW7) is a member of the RING E3 ligase family, which is a subunit of the SKP1, cullin1, and F-box protein ubiquitin ligase complex (29). Low expression and mutation of FBW7 has been frequently detected in various human tumors such as breast cancer (97), colon cancer (98), and gastric cancer (99). Therefore, FBW7 is generally considered a tumor suppressor. According to the analysis of public datasets TCGA-BLCA and GSE13507, it has been verified that the mRNA expression levels of FBW7 are significantly downregulated in bladder tumors compared with normal samples (50). Kaplan–Meier analysis suggested that patients with BCa with high FBW7 expression levels exhibited longer survival times. Collectively, these results indicate that FBW7 may serve as a tumor suppressor in bladder cancer. ZMYND8 was acted as a common oncogene in numerous tumors, including bladder cancer (50). Bioinformatics predictive analysis from the UbiBrowser platform (http://ubibrowser.ncpsb.org/) and ubiquitination assays demonstrated that in T24 cells, ZMYND8 was a substrate target of FBW7. FBW7 is a tumor suppressor that is and downregulated in BCa. Low expression of FBW7 can increase the protein levels of ZMYND8 and promote BCa progression (50). This result was further confirmed in clinical samples.

Moreover, FBW7 was verified to be an NF-κBp65 downstream effector. Through promoting RHO guanosine diphosphate dissociation inhibitor (RhoGDIα) protein degradation, FBW7 significantly inhibited BCa migration (51). Mechanistically, p65 inhibited PTEN mRNA transcription, whereas PTEN accelerated FBW7 protein degradation. This revealed the function of the p65/PTEN/FBW7/RhoGDIα axis in mediating bladder cancer migration and expands the theoretical support for the regulation of the NF-κBp65 and PTEN pathways in BCa treatment.

#### 2.2.7 MDM2

MDM2 is reported to mainly target p53 protein in various types of cancer, including bladder cancer (100). The SNP309 polymorphisms of MDM2 is associated with an improved survival rate of bladder cancer (101). MDM2 is upregulated by the OCT3/4/TET1/NRF2 axis, which contributes to increased immune escape in bladder cancer (102). Amounts of inhibitors, such as MDM2 exerted an influence on immunity in the tumor microenvironment, such as APG-115 and AMG-232. APG-115 can enhance the efficacy of PD-L1 blockade (103) and AMG-232 (104) can increase the ability to kill T cells. Furthermore, gene amplification of MDM2 can act as a predictive marker for PD-L1 targeted therapy response (105).

### 2.3 Other E3 ubiquitin ligases

Several other E3 ubiquitin ligases are also involved in bladder tumorigenesis. RFWD3 is highly expressed in bladder cancer tissue and correlates with a higher N stage and poorer prognosis (58). A bladder cancer genome-wide CRISPR/Cas9 KO screen showed that HUWE1 was correlated with cisplatin sensitivity in bladder cancer; however, the underlying mechanism has not been elucidated (59). MDM2 binds to PPARγ to ubiquitinate and downregulate its PPARγ expression (60). Denticleless E3 ubiquitin protein ligase homolog (DTL) is overexpressed in BCa, and increased DTL expression correlates with malignant biological behavior and promotes BCa progression through the AKT/mTOR pathway (61). A pan-cancer study also showed that DLT could be a potential immunotherapy biomarker (106).

TRAF4 can bind to and target another E3 ligase, SMURF1, for proteasomal degradation (21). As SMURF1 is a negative regulator of the BMP/SMAD signaling pathway, TRAF4 can promote BMP/SMAD signaling and inhibit bladder cancer progression (21). TRIM21 acts as a ubiquitin E3 ligase to degrade ZHX3, which is involved in bladder cancer progression and metastasis (52). The expression level of TRIM65 is frequently upregulated and ANXA2 is ubiquitinated and degraded by TRIM65. Bladder cancer patients with low ANXA2 expression and high TRIM65 expression showed the poorest outcome (53). RITA1 recruits TRIM25 to ubiquitinate RBPJ to accelerate its degradation via the proteasome, which leads to transcriptional inhibition of Notch1 downstream targets (54). TRIM26 plays an oncogenic role in bladder cancer by regulating cell proliferation, migration, and invasion via the AKT/GSK3β/β-catenin pathway (55). CUL4B is a scaffold protein in the CUL4B–RING ubiquitin ligase (CRL4B) complexes. CUL4B levels are overexpressed and positively associated with the malignancy of BCa, and CUL4B epigenetically represses the transcription of miR-372/373 by catalyzing the monoubiquitination of H2AK119 in the gene cluster encoding miR-372/373, which further leads to the upregulation of PIK3CA and activation of AKT (56).

Reprogramming cell metabolism is a hallmark of cancer (107, 108). Aerobic glycolysis has been extensively studied in several cancers, including bladder cancer (107). It is characterized by increased glucose uptake and lactate production under normal oxygen conditions. Elevated glycolytic flux in cancer cells is mediated by glycolysis-associated signature genes, including GLUT1 (109). GLUT1 driven glycolytic reprogramming is considered necessary for tumor cell growth (110).Wang et al. identified GLUT1 as the downstream substrate of TRIM38 and TRIM38 can constrain bladder tumor progression through ubiquitination and degradation of GLUT1 (57). TRIM38 has been verified to be a predictive biomarker related to prognosis, with low expression in BCa (57).

## 3 Deubiquitinases in bladder cancer

### 3.1 Overview of deubiquitinases

Deubiquitinases (DUBs) are proteases that remove ubiquitin from substrates or cleave ubiquitin chains to regulate ubiquitination (111). It is important to regulate the processes of deubiquitination and ubiquitination (112). DUBs consist of cysteine proteases and metalloproteinases that specifically cleave ubiquitin molecules on protein substrates (113). Approximately 100 different DUBs can be broadly classified into seven distinct superfamilies (114). Six of these families are cysyrine-based DUBs, including Ub C-terminal hydrolases (UCHs), Ub-specific proteases (USPs), Machado-Josephin domain proteases (MJDs), ovarian tumor proteases (OTUs), motifs interacting with the Ub-containing novel DUB family (MINDY), zinc-finger-containing Ub peptidase (ZUP1), and Jab1/Mov34/MPN+ protease (JAMM) family members, which are zinc-binding metalloproteases (115).

Numerous studies have demonstrated that the effect of protein deubiquitination is associated with the occurrence and development of cancers, such as prostate cancer, lung cancer, stomach cancer, and bladder cancer (116–120). A summary of the deubiquitinases involved in BCa is presented in **Table 2**.

**Table 2:** Deubiquitinases in bladder cancer tumorigenesis.

DUBs	Function	Substrate	Pathway	Reference
OTUD5	Promoting/oncogene	RNF186	mTOR	(121)
OTUB1	Promoting/oncogene	ATF6α		(122)
		SLC7A11		(123)
MINDY1	Promoting/oncogene	YAP		(119)
UCHL5	Promoting/oncogene	c-Myc	AKT/mTOR	(124)
	Cisplatin resistance		β-catenin, c-Myc	(125)
USP24	Promoting/oncogene	GSDMB	GSDMB/STAT3	(126)
USP13	Tumor suppressor	PTEN		(127)
USP7	Tumor suppressor	CCDC6		(128, 129)
USP8	Promoting/oncogene	AUF1	USP8/AUF1/RhoGD1β	(130)
USP38	Tumor suppressor	METTL14		(131)
USP22 USP18,USP28	Promoting/oncogene			(132–135)

### 3.2 Roles of deubiquitinases in bladder cancer

#### 3.2.1 OTUD5

There are 16 types of cysteine protease OTU family members, including OTUB, OTUD, A20-like, and OTULIN subfamily (113). The OTUD family is one of the subfamilies including OTUD1, OTUD2/YOD1, OTUD3, OTUD4, OTUD5/DUBA, OTUD6A, OTUD6B, and ALG13 (113, 136). OTUD5 has been the focus of numerous studies and plays pivotal roles in various cellular processes. The first report of function of OTUD5 is to negatively regulate IFN-1 expression by cleaving the polyubiquitin chains on TRAF3 (137). Furthermore, OTUD5 regulates DNA damage repair, transcription, and innate immunity (138, 139).

In bladder cancer, OTUD5 has been shown that is highly expressed in tumor tissues compared with normal urothelial cells (121). OTUD5 knockdown inhibited the cell proliferation, and OTUD5 positively regulated the mTOR signaling pathway to promote cell proliferation. Specifically, OTUD5 stabilizes RNF186 by deubiquitination, leading to sestrin2 degradation, which acts as a feedback inhibitor of the mTOR signaling pathway (140, 141). Everolimus treatment, an mTOR inhibitor, with simultaneous OTUD5 knockdown seems to be an ideal strategy for bladder cancer treatment (121).

#### 3.2.2 OTUB1

The deubiquitinase OTUB1 is significantly more highly expressed in bladder cancer tumor tissues than in normal tissues (122). Kaplan–Meier survival analysis confirmed that bladder cancer patients with low OTUB1 expression had significantly superior overall survival compared to those with high OTUB1 expression. It has been found that OTUB1 can stabilize activating transcription factor 6α (ATF6α) in response to endoplasmic reticulum stress and promote bladder cancer progression (122). Numerous studies have indicated that ferroptosis is an important and independent mechanism of tumor suppression (142). Solute carrier family 7, membrane 11 (SLC7A11), a 12-pass transmembrane protein, acts as a potential biomarker for protecting cancer cells from oxidative stress and ferroptosis (143). Liu et al. discovered a distinct mechanism by which OTUB1 mediates ferroptosis in bladder cancer via the stabilization of SLC7A11 (123).

#### 3.2.3 MINDY1

MINDY1 (also known as FAM63A) has been reported that contains MIU motifs with high selectivity for binding and cleaving K48-linked polyUb (144). The Hippo signaling pathway has emerged as a critical pathway in the regulation of bladder cancer tumorigenesis, and TAZ and YAP are important effectors of this pathway (145–147). MINDY1 removes the K48-linked ubiquitin chain from YAP, thus inhibiting proteasome-mediated YAP degradation, which will in turn promote the expression of YAP downstream genes, CTGF, ANKRD1, and CYR61 (119).

#### 3.2.4 UCHL5

UCHL5 is abnormally upregulated in human cancer tissues and cell lines, such as pancreatic adenocarcinoma, gastric cancer, endometrial cancer, and bladder cancer (124, 148–150). Upregulation of the TGF signaling pathway is the main mechanism by which UCHL5 modulates malignant tumor progression (151–153). UCHL5 is overexpressed in patients with bladder cancer patients, and high expression is associated with poor prognosis and tumor progression. Mechanistically, UCHL5 activates the AKT/mTOR signaling pathway and increases c-Myc expression, which promotes tumor occurrence and progression (124). Meanwhile, it has been reported that the UCHL5 inhibitor b-AP15 suppresses bladder cancer stemness by inhibiting the β-catenin and c-Myc signaling pathways and overcomes cisplatin resistance (125). b-AP15 has been demonstrated to have synergistic effects in combination with cisplatin, gefitinib, gemcitabine, and vinorelbine in lung cancer cells (154). In bladder cancer cell lines and mouse xenograft models, b-AP15 combined with cisplatin showed superior therapeutic effects compared to cisplatin monotherapy (125). These studies indicate that UCHL5 may act as a potential therapeutic target, and that b-AP15 may be a new choice for patients with cisplatin resistance.

#### 3.2.5 USP24

Ubiquitin-specific peptidase 24 (USP24), consisting of 2,620 amino acids, serves as a deubiquitinase (155). However, the biological function of USP24 in cancer is poorly understood. It has been reported that USP24 binds to GSDMB to deubiquitinate and stabilize GSDMB. GSDMB promotes cancer cell growth by activating STAT3, which increases the expression of HK2, LDNA, ENO2, and IGFBP3 to enhance glycolysis in bladder cancer cells (126). EOAI3402143, a USP24 inhibitor, can block this process, which provides a therapeutic strategy for inhibiting the GSDMB/STAT3 axis (126).

#### 3.2.6 USP13

USP13 belongs to the Ub-specific protease subfamily of deubiquitinase family. USP13 has been indicated in suppressing tumor occurrence by deubiquitinating anti-oncogenes, including p53 (156), PTEN (157), and MITF (158), and subsequently stabilizing these proteins. As mentioned above, PTEN acts as a key tumor suppressor in bladder cancer via inhibition of the PI3K/AKT/mTOR signaling pathway. Otherwise, NF-κB activation has been reported to be essential for inhibition of PTEN expression (159, 160). PTEN is deubiquitinated by USP13 in bladder cancer, and its stabilized expression suppresses tumor progression (127). There is also a potential regulatory loop in which NF-κB induces miR-130b/301b overexpression, decreasing USP13 expression and subsequently leading to the downregulation of PTEN overexpression (127).

#### 3.2.7 USP2a/7/8/18/22/28/38

Several studies have demonstrated that other USPs serve as oncogenes in BCa tumorigenesis (128, 130, 132–134, 161). Jeong et al. detect the mRNA expression of USP2a in bladder cancer tissues and normal tissues. The results indicate that the expression of USP2a in bladder cancer is downregulated and that high stage muscle invasive bladder cancer (MIBC) has lower USP2a expression. USP2a can be specifically used as a potential marker to stratify the more invasive phenotype of MIBC (132).

USP7 has been reported to modulate CCDC6 levels in bladder cancer and lung neuroendocrine cancers (129). CCDC6 acts as a tumor suppressor, its deficiency determines the sensitivity of PARP-inhibitors (162, 163). In a recent study, P5091, an inhibitor of USP7, promoted CCDC6 degradation and sensitized bladder cancer cells to the cytotoxic effect of the PARP-inhibitor olaparib (128).

The non-canonical NF-κB subunit p52 upregulates USP8 expression at the transcriptional level, and USP8 modulates AUF1 protein degradation. USP8 plays a significant role in the p52/miR-145/Sp1/USP8/AUF1/RhoGD1β axis, which can act as a positive regulator of bladder cancer invasion (130).

USP22 is a positive regulator of tumor growth. Silencing USP22 by interfering with RNA inhibits proliferation and induces cell cycle arrest in BCa cells (133). USP18 and USP28 have been reported to serve as prognostic markers for bladder cancer (134, 135). A study also revealed a feedback loop of USP38 and METTL14 in bladder cancer to suppress BCa progression. METTL14 stabilizes USP38 mRNA expression through YTHDF2-dependant m6A modification and USP38 enhances the stability of METTL14 by deubiquitination of METTL14 (131).

## 4 Role of E3 ligases and DUBs in immunotherapy of bladder cancer

The concept of immunotherapies for bladder cancer can be divided into cytokine-based treatment, genetically engineered immune cells (adoptive cell therapy), oncolytic viruses, bispecific antibodies, intravesical therapy with Bacillus Calmette–Guerin (BCG) vaccine, immune checkpoint inhibitors (ICIs), and antibody–drug conjugates (ADCs) (10, 164, 165).

BCG immunotherapy remains the gold standard treatment for patients with non-muscle-invasive bladder cancer (NMIBC) at a high risk of progression or recurrence (166). Although it has been used in clinical practice since 1976, the mechanism of the BCG vaccine in BCa is not completely understood. Upon attachment to the urothelium and internalization, it is thought to induce innate and adaptive immune responses. However, whether a combination of reagents targeting E3 ligases or DUBs can augment the response to BCG or conquer certain patients’ unresponsiveness to BCG warrants further exploration (167).

The adoption of ICIs in bladder cancer has dramatically changed its treatment landscape (168). ICIs are now approved for the treatment of BCa at all stages, depending on the specific tumor characteristics (10). Immune checkpoint inhibitors can enhance T-cell responses and provide promising clinical outcomes in bladder cancer. However, this treatment strategy has only a 13%–24% response rate among patients with bladder cancer. A deeper exploration of the mechanisms that regulate PD-1/PD-L1 expression and stability may help increase clinical effectiveness. During the last decade, intensive evidence has demonstrated that PD-1/PD-L1 protein expression is regulated by the ubiquitin-mediated proteasome degradation pathway (169–172).

**Figure 1:**
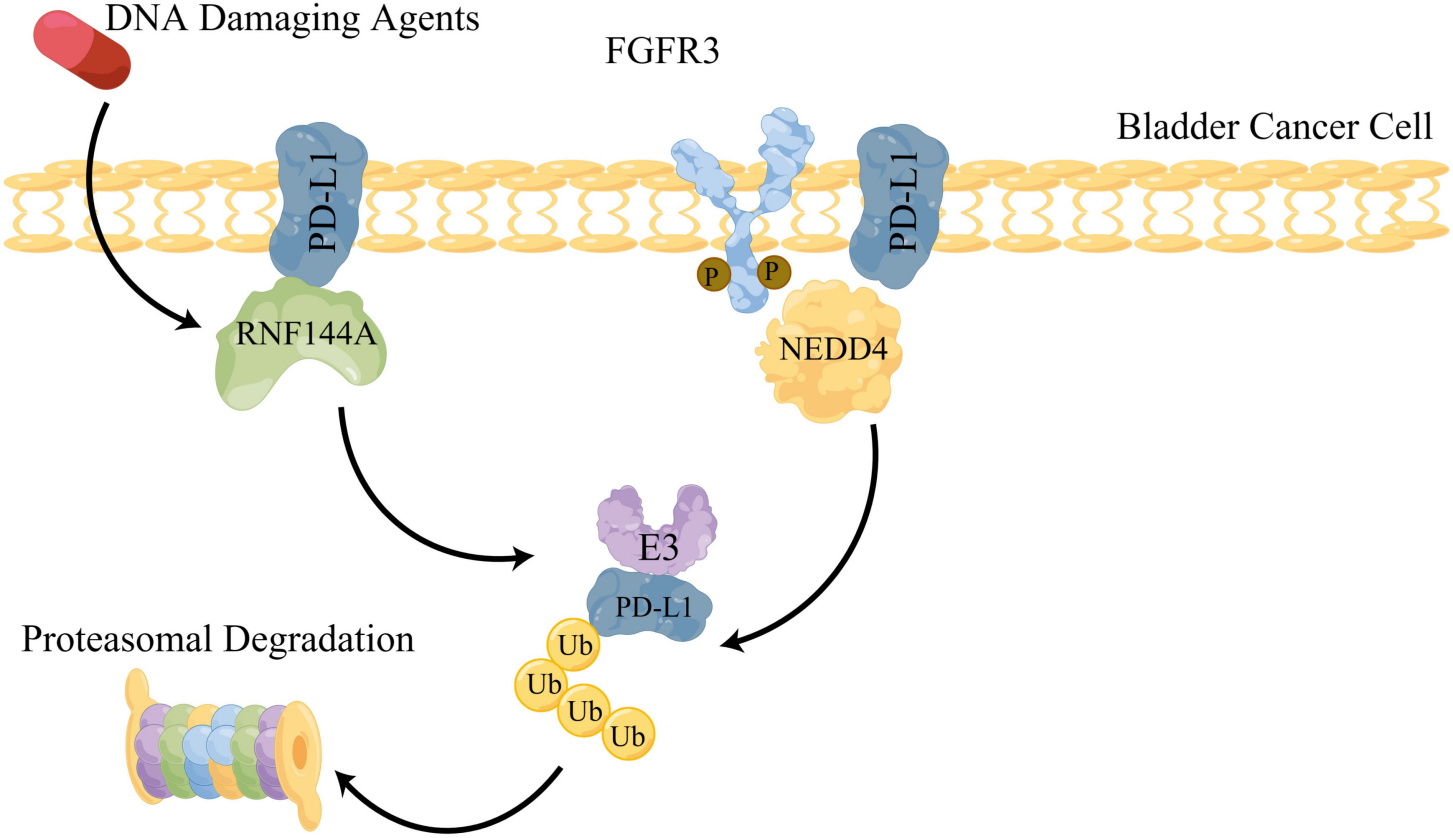
Graphic model of interaction between PD-L1 and E3 ligases in bladder cancer.

RNF144A and NEDD4 have been reported to participate in the regulation of PD-L1 expression (**Figure 1**). The basal-squamous subtype of bladder cancer expresses relatively low levels of RNF144A and high levels of immune checkpoint protein programmed cell death ligand-1 (PD-L1) (41). The carboxyl-terminal region (aa 250–292) of RNF144A is responsible for its interaction with PD-L1 and RNF114A mainly targets glycosylated PD-L1 for degradation (40). PD-L1, primarily in the insoluble fraction, interacts with RNF144A, which contains the plasma membrane and intracellular vesicles (40). RNF114A knockout stabilizes PD-L1 and leads to a reduction in tumor-infiltrating CD8+ T-cell populations in BBN-induced bladder tumors (40). Thus, RNF144A E3 ligase may be a promising therapeutic target for immunotherapy or combined therapy.

FGFR3 is an eligible target for the treatment of bladder cancer. p-FGFR3 and NEDD4 co-localized at the cell surface of bladder cancer cells. It has been demonstrated that NEDD4 can be phosphorylated to greatly improve its ubiquitination capacity by FGFR3 (16). NEDD4 depletion using CRISPR/Cas9-sgRNA remarkably upregulated PD-L1 expression in bladder cancer cells. NEDD4 targets and catalyzes the K48-linked polyubiquitination of PD-L1. These results revealed that NEDD4 is a critical regulator of PD-L1 expression in bladder cancer with FGFR3 activation (16). Thus, NEDD4 E3 ligase may be a promising therapeutic target in the bladder with immunotherapy or combined therapy.

USP7 has been shown to regulate anti-tumor immune responses. The activity of Treg cells is impeded by its inhibitor and the polarization of tumor-related macrophages is enhanced (173). One study reported that USP7 expression is positively related to PD-L1 expression and USP7 directly binds to PD-L1 which stabilized it in gastric cancer (117).However, the function of USP7 inhibitors in enhancing the immune response in bladder cancer remains unclear. Therefore, it is essential to investigate the role of USP7 in bladder cancer.

Although some other DUBs, including USP22 (174) and USP9X (175), have been shown to regulate PD-1/PD-L1 expression, no research has been conducted on bladder cancer. Because ubiquitination or deubiquitination of certain molecules can be cellular context-dependent, E3 ligases and DUBs targeting PD-1/PD-L1 in other tumors should be further verified in bladder cancer. Several E3 ligases and DUBs, especially DUBs, can be directly targeted by small molecular drugs; thus the combination of specific inhibitors and ICIs might be attractive and promising for enhancing ICI treatment effects (176). Notably, deubiquitinating enzymes are potential biomarkers for treatment selection and prognosis prediction (177).

In addition to PD-1 or PD-L1 based immunotherapy, antibody–drug conjugates (ADCs) have recently shown great progress. An ADC targeting nectin-4 (Enfortumab Vedotin) has shown significantly prolonged survival in patients with locally advanced or metastatic urothelial carcinoma who previously received platinum-containing chemotherapy and progressed after treatment with a PD-1 or PD-L1 inhibitor (178). For patients who are not eligible for cisplatin-containing chemotherapy, Enfortumab Vedotin Plus Pembrolizumab may be a safe and effective surrogate for previously untreated advanced bladder cancer patients (179, 180). Nectin-4 is a transmembrane protein overexpressed in bladder cancer and several other malignancies, making it an appropriate target antigen for ADCs. However, little is known about its role in tumor development, progression, and immunomodulatory functions. It might also be interesting to investigate the regulation of stabilization and degradation (180).

Casitas B lymphoma-b (Cbl-b) is an E3 ligase that can modulate PD-L1 ubiquitination and degradation after inhibition of PI3K/Akt, Jak/Stat, and MAPK-Erk signaling (181). Cbl-b can also target the ubiquitination of PI3K NEDD4, PLCγ, and the zeta-subunit of TCR. Interestingly, Cbl-b also serves as a downstream regulator of both CD28 and CTLA-4 signaling pathways. Thus, both innate and adaptive immune cells are regulated by E3 ubiquitin ligase, promoting an immunosuppressive tumor microenvironment. This implicated a complex regulatory loop between CTLA-4, E3 ligase Cbl-b, and PD-L1. Novel Cbl-b inhibitors offer antigen-specific immune stimulation and are promising therapeutic tools in the field of immune-oncology (182).

## 5 Summary and perspectives

In summary, patients with advanced bladder cancer have poor survival rates, and immunotherapy may be a promising method for these patients. The use of single-agent immunotherapy or combined immunotherapy may be a further direction for treating advanced bladder cancer. A better understanding of bladder cancer progression and its regulation of immune-related molecules will help us to develop better therapeutic drugs and select appropriate patients. However, the overall efficacy is unsatisfactory, and a large number of patients cannot benefit from these agents due to a lack of response. PTMs have been indicted to play a significant role in the regulation of protein stabilization of the PD-1/PD-L1 axis. The ubiquitinase–protease system plays a pivotal role in bladder cancer, including in tumor progression, cisplatin resistance, tumor suppression, and predictive biomarkers. Notably, numerous E3 ligases and DUBs act as oncogenes, including RBX1, cIAP2, CUL4B, OTUD5, MINDY1, and USP24. FBW7, USP13, USP2a, USP8, and USP7 serve as tumor suppressors. Furthermore, emerging evidence has demonstrated that RNF114A and NEDD4 can modulate PD-L1 ubiquitination, which in turn leads to the subsequent modulation of immunosuppression and anticancer effects.

This review highlights the significant role of the UPS in bladder cancer carcinogenesis and in the regulation of certain immune therapy-related molecules, including PD-1/PD-L1. These findings indicate that E3 ligases and DUBs may act as potential targets for bladder cancer therapy or a promising therapeutic approach to promote immunotherapy effectiveness by regulating ubiquitination and deubiquitination.

## Author contributions

MW, ZZ, and ZL wrote the manuscript and YZ and CX edited it. All authors contributed to the article and approved the submitted version.
